# Randomized feasibility trial to assess tolerance and clinical effects of lithium in progressive multiple sclerosis

**DOI:** 10.1016/j.heliyon.2020.e04528

**Published:** 2020-07-28

**Authors:** John R. Rinker, William R. Meador, Peter King

**Affiliations:** a Department of Neurology, University of Alabama at Birmingham, 1720 7^th^ Avenue South, Birmingham, AL, 35294, USA; b Birmingham VA Medical Center, 700 19^th^ Street South, Birmingham, AL, 35233, USA

**Keywords:** Neurology, Nervous system, Immunology, Immune disorder, Clinical research, Progressive multiple sclerosis, Lithium, Clinical trial

## Abstract

**Background:**

Disability accumulation in progressive multiple sclerosis (MS) results from inflammatory and neurodegenerative mechanisms. In animal models of MS, lithium acts to reduce inflammatory demyelination, and in models of neurodegenerative diseases, lithium also slows neuronal death. Prospective studies of lithium in MS patients have not been previously undertaken.

**Objective:**

To determine the tolerance and feasibility of using low-dose (150–300 mg/daily) lithium as a pharmaceutical intervention in a cohort of subjects with progressive MS, and to gauge preliminary effects of lithium on change in brain volume over time.

**Methods:**

Patients with primary or secondary progressive MS were recruited into a 2-year, single-blind crossover trial in which subjects were randomly assigned to take lithium in year 1 or 2. The primary outcomes of interest were tolerance of lithium and percentage brain volume change (PBVC) on vs. off lithium. Secondary outcomes included relapse rates, disability changes, and self-report scales assessing fatigue, mood, and quality of life (QOL).

**Results:**

Of 24 screened patients, 23 were randomized to take lithium during year 1 (n = 11) or 2 (n = 12). Two subjects discontinued the trial due to lithium side effects. Other reasons for discontinuation included personal reasons (n = 2), worsening MS (n = 1), and development of multiple myeloma (n = 1). For the 17 who completed the trial, change in PBVC on lithium (+0.107) did not significantly differ from the observation period (-0.355, p = 0.346). Disability measured by Expanded Disability Status Scale and MS Functional Composite did not differ by lithium treatment status. On patient reported measures of mental well-being, subjects reported fewer depressive symptoms on the Beck Depression Inventory (12.3 vs. 15.8, p = 0.016) and more favorably on the mental domains of the MSQOL inventory (56.7 vs. 52.4, p = 0.028).

**Conclusions:**

Low-dose lithium is well tolerated in persons with MS. Taking lithium did not result in differences in PBVC, relapses, or disability, but conclusions were limited by study design and sample size. Despite concern for lithium-associated neurological side effects, subjects taking lithium did not report worsened fatigue or physical well-being. On measures of mood and mental health QOL, subjects scored more favorably while taking lithium.

**Clinicaltrials.gov identifier:**

NCT01259388.

## 1 Introduction

Currently approved disease modifying therapies (DMTs) for multiple sclerosis (MS) protect the central nervous system (CNS) against bouts of inflammatory demyelination. Fewer drugs slow the insidious accumulation of impairment known as progression (Montalban et al., 2017; Hartung et al., 2002; Kappos et al., 2018). The failure of most DMTs to slow progression supports the contention that progression is driven by inflammation-independent neurodegeneration (DeLuca et al., 2004), or by chronic inflammation sequestered behind the blood-brain barrier (Stadelmann and Brueck, 2008). The accumulation of clinical impairment correlates radiologically with accelerated brain atrophy, making it a suitable surrogate for neurodegeneration (Furby et al., 2008).

Lithium is a pharmacologically active cation used since the mid-twentieth century as a mood stabilizer for treatment of bipolar disorder (Mitchell and Hadzi-Pavlovic, 2000). Despite its long history of therapeutic use, lithium's mechanisms of action have only recently become elucidated, and hold promise for both inflammatory and neurodegenerative diseases. In animal models of neurodegeneration, lithium protects against glutamate excitotoxicity via activation of Akt-1 (Chalecka-Franaszek and Chuant, 1999), which inhibits apoptosis and promotes cell survival. Lithium also depletes intracellular inositol through inhibition of inositol monophosphatase, which in turn promotes synapse formation while increasing intracellular autophagy of toxic intracellular inclusions (Kim and Thayer, 2009). This mechanism was cited to explain the ameliorative effects of lithium in an animal model of amyotrophic lateral sclerosis (Fornai et al., 2008). Lithium also inhibits the constitutively active enzyme glycogen synthase kinase 3-beta (GSK3b), which itself regulates multiple transcription factors involved in regulation of apoptosis (Jope and Johnson, 2004). Inhibiting GSK3b also prevents tau phosphorylation in mouse models of Alzheimer's disease (Hampel et al., 2019).

By inhibiting GSK3b, lithium exerts immune modulating effects in two murine models of experimental autoimmune encephalomyelitis (EAE) (De Sarno et al., 2008). Coupling the potential neuroprotective mechanisms discussed above with lithium's widespread distribution throughout the CNS makes it an attractive candidate to target both the inflammatory and neurodegenerative aspects of MS.

Although lithium possesses multiple appealing qualities such as oral administration, low cost, and a well-described safety and toxicity profile, it also has a narrow therapeutic index, a propensity for neurological side effects, and unknown tolerability among MS patients. Thus, we conducted a pilot trial to assess the tolerability and feasibility of lithium in progressive MS, and to assess for preliminary evidence of efficacy using both radiological and clinical outcomes.

## 2 Materials and methods

### 2.1 Study design

This pilot study was designed as a 2-year, open-label, examiner-blinded crossover trial of low-dose lithium for patients with primary progressive (PP) or secondary progressive (SP) MS. Each subject spent one year taking lithium and one year in observation. The sequence of lithium vs. observation was determined at randomization. Principal aims of the trial were two-fold: 1) Determine the feasibility of treating progressive MS with lithium; and 2) Compare rates of change in brain volume between on- and off-lithium treatment phases. In order to maximize the number of subjects exposed to lithium and to balance the distribution of subjects between first- and second-year lithium exposure, a randomized crossover design was utilized. This also allowed for paired statistical analyses, increasing statistical power with a small number of participants.

Study procedures were completed at the Birmingham Veterans Affairs Medical Center (BVAMC), Birmingham, AL, except for MRIs, which were completed at the University of Alabama at Birmingham (UAB), Birmingham, AL. The first subject consented to participate in May 2011 and the final subject concluded the study in August 2015.

### 2.2 Participants

Requirements for consenting subjects included age from 30 to 65 years, progressive MS as defined by the 2005 revised McDonald criteria (Polman et al., 2005), and an Expanded Disability Status Scale (EDSS) (Kurtzke, 1983) score between 3 and 6.5, inclusive. Exclusion criteria included recent (within 1 month) relapse or corticosteroid treatment, any past cytotoxic therapy, medical conditions or behaviors known to increase the risk of lithium toxicity (kidney dysfunction, cardiac arrhythmias, unstable psychiatric illness, seizures, substance abuse, concurrent use of antipsychotics, diuretics, digoxin, iodide salts, or frequent non-steroidal anti-inflammatory drugs), patients with active psoriasis, and patients unable to complete study procedures.

Subjects could have either a PP or SP course, defined as prospective or retrospective neurologic worsening over one year's time in the absence of relapses. The decision to recruit both PP and SPMS subjects was based on growing evidence from natural history studies that MS progression is similar whether or not it is preceded by a relapsing course (Kremenchutzky et al., 2006; Confavreux et al., 2000), and because the focus of this trial was on the progressive rather than the relapsing attributes of MS. Subjects with relapses or steroid exposure within the previous month were excluded, as were subjects who received past cytotoxic drugs. Due to safety concerns, pregnancy and medical conditions which increased the risk of lithium usage were also excluded, and women of childbearing potential were required to affirm use of birth control. Continuation on approved MS DMTs during trial participation was permitted. Subjects were initially recruited solely from the BVAMC and later also from the UAB MS clinic due to slowing recruitment from the BVAMC.

#### 2.2.1 Randomization

Subjects were randomized using randomly mixed permuted blocks of 2 and 4 subjects, to ensure equal allocation of subjects to taking lithium in the first and second study years.

### 2.3 Approvals and consents

This study was approved by the BVAMC Institutional Review Board. Written informed consent was obtained from all participants. The study was registered on ClinicalTrials.gov (NCT01259388) and conducted according to 2010 CONSORT guidelines (Schulz et al., 2010).

### 2.4 Study medication and dosing

The initial target dose for lithium derived from serum levels measured in mice studied in the EAE experiments referenced above. Mice fed a lithium-enriched diet with mean serum levels of approximately 0.53 mEq/L experienced protection against EAE (De Sarno et al., 2008). Since lithium side effects increase with plasma level, and since the therapeutic range for lithium for psychiatric conditions is typically 0.6–1.2 mmol/L, the target level for this study was set below the psychiatric range in hopes of minimizing side effects.

All lithium used in the trial was procured by the BVAMC Research Pharmacy as 150 mg capsules of lithium carbonate (Roxane Laboratories, Inc). At the beginning of the trial, subjects initiating lithium were begun on 150 mg twice daily then titrated to a target of 300 mg twice daily one week later. The original dosing plan was to titrate dose to maintain trough plasma levels between 0.5-0.8 mmol/L. However, due to a combination of factors including inconsistent laboratory measurements of lithium levels, logistical problems in drawing trough drug levels, and intolerance of 600 mg/day in two subjects, the study protocol was altered within the first 3 months of the trial and the target dose was lowered to 150 mg twice daily. The dosing changes began with the 5^th^ subject randomized to first-year lithium, and target plasma lithium levels were discarded as a requirement for continued study participation. All subsequent study subjects were dosed to a target of 300 mg/day, or 150 mg/day if 300 mg/day was not tolerated. Compliance was monitored by pill counts at each return visit. Plasma levels were no longer used to determine need for dose adjustment following the change from a level-based dosing strategy to a fixed dose approach.

### 2.5 Study procedures and outcomes

Comprehensive assessments occurred at baseline and at 6-month intervals for two years. Assessments at comprehensive visits were as follows: Interval medical and neurological history (treating physician); EDSS (examining physician); MS Functional Composite (MSFC) (Cohen et al., 2001) and Symbol Digit Modality Tests (SDMT) (Brochet et al., 2008) (neuropsychologist); and patient-reported outcome scales. At baseline, year 1, and year 2 time points, subjects also underwent cranial MRI. During the lithium-treatment phase of the trial, subjects also were evaluated at months 1, 3, and 9 for the purpose of laboratory monitoring and medication refills. The PI served as the treating physician throughout the study. Both the examining physician and the neuropsychologist were blind to the treatment status of the subjects (on- vs. off-lithium). Subjects did not see examining physician at months 1, 3 and 9 of the lithium-treatment year to maintain blinded status.

Due to the pilot nature of the trial, drug tolerance and feasibility were the principal aim of the research. At each study visit, subjects were questioned about common side effects of lithium by structured interview, and given the opportunity to report new or worsening neurologic symptoms. Subjects were also evaluated at each visit for changes in neurological exam and laboratory abnormalities known to be associated with lithium usage. Safety and tolerability of lithium was determined by calculating frequency of side effects and adverse events.

The second principal aim of the study was to test the hypothesis that lithium treatment would decrease the rate of brain atrophy as measured by percentage change in brain volume (PBVC) using Structural Image Evaluation, using Normalization of Atrophy (SIENA) (Smith et al., 2002) software. To test the hypothesis, subjects underwent cranial MRIs using a standardized protocol on a Philips Achieva 3T head-only MRI at baseline and at the end of each of the two trial periods (years 1 and 2). The imaging protocol followed the 2003 Consortium of MS Centers imaging guidelines (Traboulsee et al., 2003) as well as a 3D T1 sequence for use in brain volume assessments. As this was a crossover study, each subject served as his or her own control. SIENA analyses were performed by the primary investigator. Even though SIENA runs as an automated analysis, MRIs were anonymized and coded so as to minimize risk of interpreter bias.

MRIs were reviewed for incidental findings by a physician board-certified in Neuroradiology, otherwise unaffiliated with the study.

Secondary clinician-assessed outcomes included relapse frequency and changes in EDSS, MSFC, and SDMT. Patient-reported outcome measures included fatigue (Modified Fatigue Impact Scale, MFIS) (Fisk et al., 1994; Kos et al., 2003), depression (Beck Depression Inventory, BDI) (Beck et al., 1996), and quality of life (MS Quality of Life, MSQOL-54) (Vickrey et al., 1995).

Blood samples were collected and peripheral blood mononuclear cells (PBMCs) were fractionated and frozen at -80 degrees Celsius from subjects at two time points during on- and off-lithium periods. Upon completion of the trial, samples were analyzed to determine GSK3b phosphorylation and cytokine-secreting profiles of the cells, which will be reported separately.

### 2.6 Statistical methods

As this was a pilot study focused on feasibility, recruitment goals were driven by local constraints over efficacy-based power calculations. Subjects were recruited entirely from the MS clinics at the BVAMC and UAB. A power calculation was performed based on change in PBVC. In order to detect a within-subject effect size of 0.66, we determined 20 subjects would be needed to complete the study. Acknowledging the potential for drop outs, more than 20 subjects were recruited, and recruitment ended in mid-2013 knowing that the study would conclude at the end of 2015.

All statistical analyses were completed using JMP (Cary, NC). Due to the crossover design of the trial, changes in measures of brain volume and disability (EDSS, MSFC) were determined for on- and off-lithium treatment periods, then those changes were compared using paired *t*-tests (for brain volume and MSFC) or non-parametric tests (for EDSS). For patient report scales (BDI, MFIS, MSQOL-54), since these were obtained at 6 and 12 month time points, these scores were averaged and compared between trial phases using paired *t*-tests. Adverse events were reported as frequencies.

## 3 Results

### 3.1 Baseline characteristics

**Figure 1:**
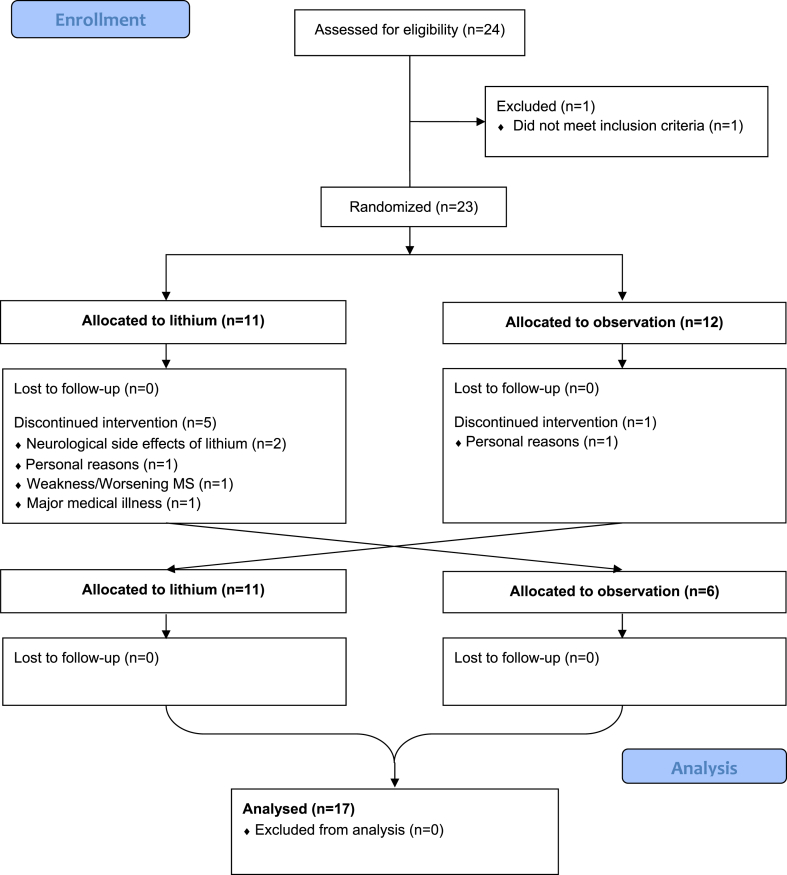
CONSORT diagram: A pilot trial of lithium in progressive multiple sclerosis.

A total of 24 subjects were recruited between May 2011 and August 2013. Recruitment, randomization, and attrition of study subjects are summarized in Figure 1. The study concluded when the final enrolled subject completed the two-year trial period in August 2015. A total of 17 subjects completed the full study.

Subject demographics are summarized in Table 1, and clinical assessments in Table 2. Groups were well balanced after randomization with respect to gender, ethnicity, pattern of MS (PP or SP), and EDSS.

**Table 1:** Subject demographics and baseline characteristics.

	Screened and randomized	Completed study
Number of subjects, n	23	17
**Demographics**		
Male, n (%)	14 (60.9)	11 (64.7)
Enrollment age, years (SD)	51.0 (7.8)	50.9 (7.6)
Age range, years	38–64	39–62
Ethnicity, n (%)		
White	15 (65.2)	12 (70.6)
Hispanic	1 (4.4)	1 (5.8)
Black	7 (30.4)	4 (23.5)
**Disease Characteristics**		
Disease duration, years (SD)	14.0 (10.5)	15.1 (11.3)
Progression duration, years (SD)	5.5 (4.9)	5.8 (5.2)
Type of MS progression		
Secondary, n (%)	20 (87.0)	16 (94.1)
Primary, n (%)	2 (8.7)	0
Progressive relapsing, n (%)	1 (4.3)	1 (5.9)
**Disease Modifying Therapy at Entry**		
Beta-interferon, n (%)	6 (26.1)	3 (17.6)
Glatiramer acetate, n (%)	5 (21.7)	4 (23.5)
Natalizumab, n (%)	9 (39.1)	8 (47.1)
None, n (%)	3 (13.0)	2 (11.8)
**Subjects relapsing within the past…**		
1 year, n (%)	6 (26.1)	5 (29.4)
3 years, n (%)	17 (73.9)	11 (88.2)

Abbreviations: MS, multiple sclerosis.

**Table 2:** Baseline clinical assessments of subjects.

	Screened and randomized	Completed study
Number of subjects, n	23	17
**Clinical Impairment Measures**		
EDSS, median (IQR)	4 (3.5, 6)	4 (3.25,6.25)
<4, n	9	6
4–5.5, n	6	5
6–6.5, n	8	6
Timed 25 Foot Walk, mean, s (SD)	13.8 (17.0)	15.6 (19.6)
9HPT Dominant Hand, mean, s (SD)	35.2 (31.6)	37.4 (36.5)
PASAT, mean number correct (SD)	41.0 (13.0)	39.9 (13.6)
SDMT, mean number correct (SD)	38.3 (12.1)	38.4 (11.8)
**Patient Report Measures**		
BDI^1^, mean (SD)	15.8 (10.2)	15.3 (9.1)
MFIS^1^, mean (SD)	49.3 (19.9)	49.7 (21.5)
MSQOL^2^ physical, mean (SD)	46.1 (19.5)	44.4 (19.5)
MSQOL^2^ mental, mean (SD)	51.2 (23.9)	50.0 (24.7)

Abbreviations: BDI, Beck depression inventory; EDSS, expanded disability status scale; 9HPT, nine-hole peg test; MFIS, modified fatigue impact scale; MSQOL, multiple sclerosis quality of life PASAT, paced auditory serial addition test; SDMT, symbol digit modality test.

1 Higher scores indicate worse symptoms.

2 Higher scores indicate better symptoms.

Twenty of 23 randomized subjects were taking an MS DMT at the start of the study. Of those not taking a DMT, two were characterized as PPMS and the third had previously taken beta-interferon as part of a clinical trial but had not continued.

### 3.2 Lithium tolerance and adverse effects

Most symptoms experienced by subjects while taking lithium were mild and did not require treatment, dose adjustment, or withdrawal from the study. Side effects are summarized in Table 3. Two subjects did experience asymptomatic increases in their TSH without changes in thyroid hormone levels and which normalized following lithium cessation at trial's end. One subject also experienced unusual dreams while on lithium which resolved when twice daily dosing was altered so that the full daily dose was taken in the morning.

**Table 3:** Adverse events during lithium treatment (n = 23).

Adverse Event	Subjects reporting AE, n	Change in lithium dose or schedule	Withdrawal from study
Excess thirst	18	0	0
Fatigue	15	1^a^	0
Polyuria	15	0	0
Weight gain	13	0	0
Weight loss	10	0	0
Tremor	10	1	0
Cognitive changes	7	1^a^	0
Muscle spasms	6	0	0
Gait impairment	5	2	2
Agitation/Anxiety	5	0	0
Worsening MS	5	1	1
Depression	4	0	0
Acne	3	0	0
Increased TSH	2	0	0
Nausea	1	0	0
Unusual dreams	1	1	0
Multiple myeloma	1	0	1

Abbreviations: AE, adverse event; MS, multiple sclerosis; TSH, thyroid stimulating hormone.

a One subject's dose was reduced due to combined complaints of fatigue and cognitive slowing.

Six randomized subjects did not complete the study. Two subjects withdrew after experiencing abrupt worsening of their gait shortly after initiating lithium, one of them early in the trial before the lithium dose was lowered to 300 mg/day. Both had worsening cerebellar findings on exam, and both returned to baseline upon cessation of lithium. One subject with a past history of a monoclonal gammopathy developed multiple myeloma during the study. He developed severe back pain from pathological vertebral fractures and was withdrawn from the study by the PI. A fourth subject randomized to lithium in year one was concerned about worsening generalized weakness and withdrew. He continued to weaken despite cessation of lithium on a trajectory consistent with his MS. The final two discontinuations were due to personal reasons unassociated with the trial.

Two serious adverse events (SAEs) occurred during the study, including the myeloma case described above. The other SAE entailed hospitalization for placement of a suprapubic catheter for longstanding urinary retention during the observation year of the trial.

### 3.3 Lithium effects on brain volume

**Figure 2:**
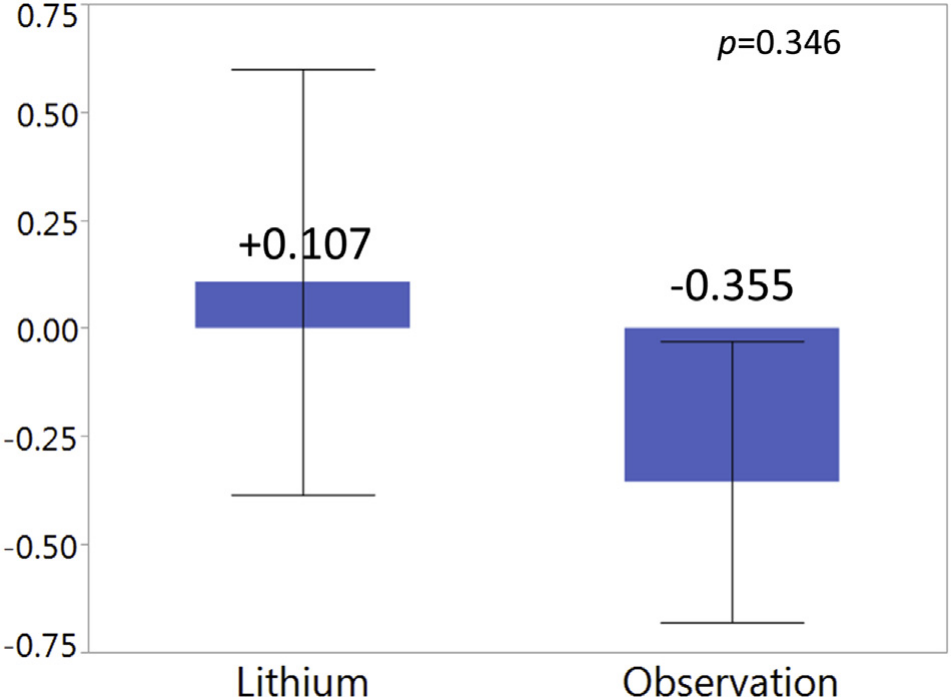
Percent change in brain parenchymal fraction between lithium and observation treatment periods.

Paired comparison of change in PBVC found a non-significant difference favoring lithium, with a slight increase in mean PBVC during the lithium treatment year (Figure 2). However, a greater than expected number of subjects experienced increases in brain volume over time, occurring in 10 subjects during lithium treatment and 7 during observation. One outlier subject experienced a 5.3% reduction in brain volume on lithium and a 4.3% reduction during observation, but for all other subjects the annual PBVC changes ranged from -1.63 (observation) to +3.04 (lithium). Excluding the outlier, the standard error around the PBVC during the observation period (0.22) was smaller than that around the lithium period (0.38), suggesting greater variability of measurement during the lithium treatment. This may suggest measurement error or a variable effect of lithium on brain volume in MS patients.

### 3.4 Secondary outcome measures

Clinical and patient-reported outcomes are summarized in Table 4. Relapses were characterized as either confirmed or suspected. Confirmed relapses required a history of acutely worsening neurologic symptoms accompanied by a change of at least 0.5 points on the EDSS or at least 1 point in a Functional System score as assessed by the blinded examining neurologist. Four relapses were confirmed, while three others were suspected. None of the on-study relapses required hospitalization, and only 3 were treated with steroids (two during the lithium-treatment phase, one during the observation phase). All the relapses involved worsening of previously experienced deficits and none were associated with development of new MRI lesions.

**Table 4:** Clinical results (n = 17).

	Observation	Lithium	Difference ±SE	*p*
**a. Clinician Derived Outcomes**				
Relapses	5	2	3	0.485^a^
Change in EDSS, median (IQR)	0 (0,0.5)	0 (0,0.25)	0	0.707^b^
EDSS unchanged, n	8	11	-	
EDSS increased, n	6	4	-	
EDSS decreased, n	3	2	-	
Δ T25FW, s^c^	2.1	0.7	1.4 ± 2.0	0.492
Δ 9HPT dominant hand, s	-1.5	0.3	1.8 ± 1.5	0.688
Δ PASAT, no. correct vs. baseline	-0.3	2.1	2.4 ± 3.7	0.521
Δ SDMT	-0.1	-1.2	1.1 ± 1.9	0.588
**b. Patient Reported Outcomes**^d^				
BDI^e^, mean	15.8	12.3	3.5 ± 1.3	0.016
MFIS^e^, mean	49.9	46.8	3.1 ± 1.9	0.125
MSQOL^f^ – Mental, mean	52.4	56.7	-4.3 ± 1.8	0.028
MSQOL^f^ – Physical, mean	40.4	43.3	-2.9 ± 2.3	0.237

Abbreviations: 9HPT, nine hold peg test; BDI, Beck depression inventory; EDSS, expanded disability status scale; MFIS, modified fatigue impact scale; MSFC, multiple sclerosis functional composite; MSQOL, multiple sclerosis quality of life PASAT, paced auditory serial addition test; SDMT, symbol digit modality test; T25FW, timed 25 foot walk.

a Fisher's exact test.

b Wilcoxan/Kruskal-Wallis.

c n = 16; one outlier removed due to poor performance on 25 F W, skewing central tendency of data.

d Results averaged from 6 and 12 month assessment time points.

e Higher scores indicate worse symptoms.

f Higher scores indicate better symptoms.

Of the two measures included to assess neurologic impairment (EDSS and MSFC), neither changed significantly during the lithium or observation phases. The majority of subjects maintained stable EDSS scores during the study, and the largest one-year change in EDSS was a 1 point increase which occurred during the observation year. Of the subjects who experienced an increase in EDSS, only one experienced an increase during both study years. MSFC scores also remained stable and not significantly different between the lithium and observation phases of the trial. The 9 Hole Peg Test times did not differ between the treatment phases, and both 25 foot walk times and PASAT scores were not significantly different but trended slightly better during the lithium treatment phase of the trial (Table 4a). SDMT scores, collected as a cognitive assessment in addition to the PASAT component of the MSFC, did not differ either on or off of lithium.

Patient-reported outcomes were included in order to capture possible effects of lithium on mood, fatigue, and overall quality of life (Table 4b). Values are averaged from the 6 and 12 month time points during each trial phase. Despite the low doses of lithium used (typical dosages for treatment of bipolar disease range from 600 to 1200 mg/day), there was a significant improvement in the BDI during lithium treatment which brought the mean BDI score below 14, which is a commonly used threshold for detecting depression (Beck et al., 1996). Five subjects reported a mean BDI above 14 while on lithium, compared to 12 during observation. Similarly, the mental score from the MSQOL improved during lithium treatment. The MSQOL physical component score and the MFIS did not differ between lithium and observation phases of the study.

## 4 Discussion

These results demonstrate that low-dose lithium carbonate may be successfully administered to patients with progressive forms of MS. However, even though there was a trend towards brain volume stabilization during lithium treatment, there is too much variability in the data to draw conclusions about possible effects of lithium on brain volume over time. While these results do not establish efficacy of lithium as a neuroprotective agent, the study results do justify development of a larger, controlled trial in which efficacy is the primary endpoint.

A major concern upon beginning the trial was whether MS patients, particularly those with fixed neurologic impairment, would tolerate lithium given its neurologic side effects such as tremor, fatigue, and cognitive slowing. These concerns initially appeared to be founded, as some early assignees to lithium therapy experienced problems with gait and balance, tremor, and fatigue. Once the daily dose was lowered from 600 to 300 mg, tolerance improved significantly and only one additional subject discontinued due to lithium intolerance in the following 3 years. In looking ahead to future studies of lithium in the MS population, side effects may be broadly grouped into three categories: 1) Side effects known to be associated with lithium (high frequency tremor, thyroid dysfunction); 2) Dose-dependent side effects which exacerbate existing MS symptoms (fatigue, cognitive slowing); and 3) Dose-independent symptoms which produce specific patterns of worsening in the MS population (gait ataxia). A blinded future trial should clarify the contribution of lithium to symptoms that overlap with MS itself, as well as identify predictive factors for the more dramatic examples of intolerance, such as gait ataxia.

The serial brain volume measurements in this progressive MS cohort suggest a possible stabilizing effect of lithium on brain volume, but differences did not rise to statistical significance, and as such should be interpreted with caution. A number of subject and study design factors may have confounded the measurement of PBVC: First, subjects were allowed to remain on MS DMTs throughout the study, while some took none at all, which may have affected results. Second, this study is underpowered to detect change in brain volume as a therapeutic outcome (Altmann et al., 2009). A larger study with placebo-controlled design, increased numbers of subjects, and longer duration of follow-up would have strengthened the statistical power of the trial, but budget constraints and the feasibility focus of the trial steered the imaging portion of the trial towards gathering preliminary data rather than powering to a more robust outcome measure. The effect of a treatment on brain volume also requires longer duration of follow up, which has been done with studies of existing MS DMTs (Hardmeier et al., 2005). In other words, a neuroprotective effect from a medication may result in neuronal preservation only measurable over long-term observation. Third, although lithium has a short half-life in the human body and is rapidly cleared by the kidneys, it is not known whether long-term use of lithium may effect longer-lasting changes in brain volume. Such a delayed effect could potentially have affected the measured changes in brain volume in this trial, particularly for subjects who took lithium in year two of the study.

Should the protective effects of lithium on brain volume be reproduced, additional important questions to ask should also focus on the mechanisms by which brain volume may stabilize or increase. At least one study has demonstrated a short-term gain in cortical gray matter volume among bipolar patients started on lithium (Bearden et al., 2007; Sassi et al., 2002), but it is not clear whether the volume increase is due to axonal sprouting and synapse formation, or tissue-specific changes in fluid content. A more refined approach to image analysis may shed additional light on the possible mechanisms of whole brain or even gray matter-selective increases in volume, and whether such changes are likely to represent disease-relevant neuroprotection or regeneration. Pharmaceutical effects on brain volume in MS are not a new concern. Adminstration of corticosteroids or certain MS DMTs may actually reduce brain volume by virtue of decreasing overall inflammation or brain fluid content, a process called pseudoatrophy (Zivadinov et al., 2008). Whether lithium exerts its own effects on brain volume in MS remains unclear, and could be tested in a more robust trial with fewer confounders.

The secondary, clinical outcomes collected for this trial were intended to provide additional data on safety and tolerance of lithium. Due to the short duration of the trial, differences in impairment as measured on EDSS or MSFC were not anticipated, and the MFIS and the MSQOL-54 were included to capture any lithium-related increases in fatigue or global declines in QOL. Results from the study showed no effect of lithium treatment on the clinician-assessed disability measures, on fatigue, or on physical QOL. Lithium did seem to produce a benefit on mood and mental QOL, despite being used at dosages well below those customarily used to treat psychiatric disorders. These findings suggest lithium may provide symptomatic benefits for MS patients with psychiatric symptoms, but will require additional study to further demonstrate these effects.

An important question to answer in future prospective trials is whether the dose of lithium may be kept low while still achieving meaningful therapeutic endpoints. The results from this trial suggest there may be both symptomatic and possibly neuroprotective benefits to be gained from low-dose lithium. Meanwhile, a growing body of research has found that even very low levels of lithium dosed deliberately or measured in community-dwelling persons may have important benefits in mental and neurological health (Wilson et al., 2020; Karimi et al., 2017).

In summary, this pilot trial successfully demonstrates that low-dose lithium is a viable option for study in the progressive MS population, and that the drug is safe and reasonably well-tolerated. A larger, follow-up phase 2 trial is warranted to evaluate whether neuroprotective effects can be demonstrated, and whether symptomatic effects can be reproduced.

## Declarations

### Author contribution statement

J. Rinker: Conceived and designed the experiments; Performed the experiments; Analyzed and interpreted the data; Wrote the paper.

P. King: Conceived and designed the experiments; Wrote the paper.

W. Meador: Performed the experiments; Wrote the paper.

### Funding statement

This work was supported by a Career Development Award from the 10.13039/100000738Department of Veterans Affairs (CDA2-003-10S).

### Competing interest statement

The authors declare no conflict of interest.

### Additional information

The clinical trial described in this paper was registered at ClinicalTrials.gov under the registration number NCT01259388.
